# Contributions of Work-Related Stress and Emotional Intelligence to Teacher Engagement: Additive and Interactive Effects

**DOI:** 10.3390/ijerph14101156

**Published:** 2017-09-29

**Authors:** Sergio Mérida-López, Natalio Extremera, Lourdes Rey

**Affiliations:** 1 Department of Social Psychology, Faculty of Psychology, University of Málaga, 29071 Málaga, Spain; sergioml@uma.es; 2 Department of Personality Assessment and Psychological Treatment, Faculty of Psychology, University of Málaga, 29071 Málaga, Spain; lrey@uma.es

**Keywords:** engagement, work-related stress, role ambiguity, role conflict, emotional intelligence, teachers, interaction

## Abstract

This study examined the additive and interactive effects of role stress and emotional intelligence for predicting engagement among 288 teachers. Emotional intelligence and engagement were positively associated. Role ambiguity and role conflict showed negative associations with vigor and dedication scores. The interaction of role ambiguity and emotional intelligence was significant in explaining engagement dimensions. Similar results were found considering overall teacher engagement. Emotional intelligence boosted engagement when the levels of role ambiguity were higher. Our findings suggest the need for future research examining the impact of job hindrances on the links between emotional intelligence and teachers’ occupational well-being indicators. Finally, the implications for emotional intelligence training in education are discussed.

## 1. Introduction

While teacher stress and burnout have constituted particularly relevant topics in educational research during recent decades [1,2], an approach focused on positive outcomes in organizations has been flourishing recently [3,4,5]. One dimension that has generated considerable interest among the different constructs grouped into the field of Positive Organizational Psychology is work engagement [6,7,8]. This construct is defined as a “positive, fulfilling and work-related state of mind that is characterised by vigor, dedication and absorption” [9,10]. Additionally, its discriminant validity, in relation to other constructs related to occupational well-being, has been extensively reported [6,11].

Although some authors have discussed the potential “dark” side of engagement [12], there is a growing consensus on the positive psychological and vocational impact of this dimension [6,13]. Indeed, accumulating empirical research has shown that work engagement is negatively related to health problems [12], turnover intentions [10], or withdrawal behavior [14]. Additionally, previous studies have shown that engagement is positively associated with performance [15], job satisfaction [16], and organizational commitment [5]. Furthermore, beyond its negative relationship with teacher burnout, the correlates of engagement have been widely examined in educational settings [5,17]. Previous studies have shown positive associations between teacher engagement and classroom achievement [18] or students’ engagement [19]. Moreover, this construct influences attitudes toward teaching, including organizational commitment [5,20], job satisfaction [21,22], organizational citizenship behavior [23], and workplace participation [24].

### 1.1. Role Stress 

In light of the Job Demands–Resources model [6,25], there is a growing consensus on the influence of work environment characteristics on engagement [26,27,28]. Certain job demands such as role ambiguity and role conflict have constituted an active field of research in educational settings [29,30]. Role ambiguity has been described as a type of inadequacy in cases in which clear information is not present and communication is lacking, whereas role conflict is defined as the simultaneous occurrence of two or more role pressures so that compliance with one makes it more difficult to comply with the other [31]. Meta-analytic reviews have provided extensive evidence on the impact of role ambiguity and role conflict in terms of psychological distress, poor mental health, and reduced job performance [32,33,34]. In Spain, work-related stress is considered a major concern as several studies have shown its negative associations with psychological distress in teaching professionals [17,27]. 

Regarding school organizations, role ambiguity and role conflict have been associated with a variety of deleterious effects on job satisfaction, occupational commitment, burnout syndrome [28,35], or turnover intentions [5,36]. Similarly, it has been consistently reported that role stress diminishes engagement [16,37,38,39,40]. Despite these previous findings following the JD-R (Job Demands-Resources) model [41,42,43], to our knowledge no study has explored the interactive effects of crucial work-related stressors such as role stress and EI (emotional intelligence) in the prediction of teacher engagement. Additionally, individuals do show marked individual differences in experiencing engagement at work [6]. In this sense, personal resources have been found to enhance engagement [4,22]. In particular, one dimension that has consistently gained attention in this field is Emotional Intelligence [44,45].

### 1.2. Emotional Intelligence

Emotional Intelligence (EI) has been suggested to be a crucial factor for enhancing occupational health and well-being [44,45,46]. According to Mayer and Salovey’s (1997) theoretical approach, EI is conceptualised as the capacity to perceive, assimilate, understand, and manage emotions in oneself and others [47]. While several ways of assessing EI have been developed [45], self-report measures that provide a score on perceived EI are most typically used instead of performance-based tests [48]. The construct EI has shown robust associations with a number of psychological outcomes, including health [49,50], well-being [51], job performance [52], and work attitudes [53]. Similarly, there is a growing body of research on the associations between EI and engagement [54,55,56]. On this connection, prior research has shown positive associations between EI and engagement using the same instruments as in the present study [57,58]. 

A large body of literature has shown that EI is a major resource for teaching professionals due to its associations with burnout [59] and psychological distress [60]. In fact, EI is increasingly playing a crucial role in teachers’ occupational health models [61]. Some researchers have argued that the extent to which teachers believe they possess adequate emotional skills to cope successfully with school stress is a key determinant of their affective responses [62,63]. Consistently, EI is related to higher satisfaction with life [64], increased teaching satisfaction [65], and more positive attitudes toward teaching [59]. Although little attention has been paid to EI as a moderator for the association between role stress and positive organizational outcomes, a few studies show support for this moderating role of EI. In this line, a recent study has shown negative associations between teachers’ emotion-regulation ability and work-related stress [60]. Moreover, the authors reported the interplay of both personal and organizational factors to predict unique variance of teachers’ depressive symptomatology. Lastly, positive links between EI and teacher engagement have been consistently reported [66,67]. From EI theory, personal resources such as EI may serve a moderating function through direct effects on the way individuals appraise and deal with a threatening situation or implementing changes in problem-solving behaviors. Therefore, these emotional resources may lead individuals to handle threats more constructively and thus experience reactions that are more positive at work [46]. Furthermore, EI is considered an antecedent of work engagement [6]. Consistent with the JD-R model, social and personal resources such as EI would moderate the associations between job demands and organizational outcomes. Accordingly, personal resources such as EI might energize employees, encourage their persistence, and make them focus on their efforts. In other words, these emotional resources might foster engagement in terms of vigor (energy), dedication (persistence), and absorption (focus) [6,11,25]. 

### 1.3. The Present Study

Taking into consideration the JD-R model as a valid framework for research on teacher well-being [61], there are some compelling reasons for us to consider EI and role ambiguity/conflict as concurrent predictors of teacher engagement. Firstly, the correlations of EI and role stress are only moderate [60,68]. Secondly, engagement has been found to be predicted by EI [57,67] and role stress [39,40] in diverse samples including teachers. Third, prior research aimed at examining the moderating effect of job demands in the personal resources-engagement association [20]. Therefore, we may provisionally expect to find that EI and role ambiguity/role conflict might show interactive effects for predicting engagement scores beyond the independent effects.

Although the one-dimensional structure of work engagement has become a commonly used paradigm, confirmatory factor analyses typically show that the three-factor structure of the UWES is superior to the unidimensional model that assumes engagement as a homogeneous construct, represented by an undifferentiated total score [69]. Additionally, the dimensions of work engagement are different from each other and from other organizational outcomes in that they refer to different psychological processes such as motivation (dedication), cognition (absorption), and affect (vigor). Accordingly, in this study, we aimed to provide evidence on the specific pattern of role stress and EI with engagement and, hence, to offer additional information comparing the total score and its dimensions. Therefore, we examined the potential interactive effect of role stress and EI to predict both overall engagement and its three subscales. Information about what specific aspects of teacher engagement are increased by EI alone and in combination with role stressors could provide greater insight into the nature of organizational and personal characteristics that contribute to explaining the specific dimensions of teacher engagement. Such knowledge would help researchers and practitioners developing more effective intervention programmes for teachers.

In light of the aforementioned conceptual and empirical considerations, the present study aimed to broaden the current understanding of teacher engagement by examining the unique and interactive contributions of EI and role stress. The purpose of this work was threefold. Firstly, we aimed to explore the associations between EI, role ambiguity, role conflict, and engagement in a teacher sample. Secondly, we examined the direct effects of EI, role ambiguity, and role conflict on engagement. Finally, we expected to find empirical support for the interactions between EI and role conflict/ambiguity in predicting engagement scores.

## 2. Methods

### 2.1. Participants and Procedure

Our study sample consisted of 288 teaching professionals (64.2% female) working at a variety of grade levels at public schools in Málaga. This sample was comprised of 12 elementary teachers (100% females), who taught pupils from three to five years old; 165 intermediate teachers (78.2% females), who taught students from six to 11 years old; 102 secondary teachers (56.9% males), who taught students from 12 to 17 years old; and nine teachers (66.7% males), who taught at the University of Málaga. The mean age was 41.67 years (SD = 9.80; range 22–64 years). The marital status of the participants was 28.1% single, 55.9% married, 6.9% divorced, 1.7% widow/widower, and 4.2% in a couple. Their teaching experience ranged from seven months to 41 years (Mean = 14.14 years; SD = 10.20 years). The original sample comprised 288 teachers, although most of the data reported here are based on responses ranging from 266 to 288 participants due to missing data. Missing data were replaced using multiple imputation in SPSS 22 (IBM, Armonk, NY, USA). The analyses were conducted with five replications, and results pooled across the five imputations are shown. Given our sample size, we had a sufficient power effect (0.79) to detect a small effect size in our intended analyses.

The participants were recruited with the assistance of psychology students at the University of Málaga by means of an incidental sampling. In this sense, the centers were contacted, informed about the research, and asked to participate. The participants were given a brief introduction to the project and were fully informed about the basis of their participation. No compensation was awarded to the participants. We administered the questionnaires in paper-and-pencil format with written instructions. The questionnaires were completed either at home or in small groups in their staff room under the supervision of a research assistant. The average time participants spent on completing questionnaires was about 25 min. Once the questionnaires were completed, they were returned to the research staff. The data were collected, taking into account the instructions from researchers regarding anonymity and with the informed consent of all participants. The study protocol was approved as part of Project PSI2012-38813 by the Research Ethics Committee of the University of Málaga.

### 2.2. Instruments

#### 2.2.1. Emotional Intelligence

We used the Wong and Law Emotional Intelligence Scale (WLEIS; [70]). This scale is comprised of four dimensions: appraisal of self-emotions (SEA), appraisal of others’ emotions (OEA), use of emotion on cognition (UOE), and regulation of emotion (ROE). This 16-item instrument uses a seven-point Likert-type scale and requires participants to report on a scale from (1) “totally disagree” to (7) “totally agree”. The scale has shown adequate internal consistency and evidence of validity [70]. As in prior research, we combined the subscales into a global EI score due to our interest in the overall construct [71]. The instrument was professionally translated from English into Spanish using the method of back-translation. In our study, the Cronbach’s alpha for the total EI was 0.91.

#### 2.2.2. Role Stress

Role ambiguity and role conflict were assessed with the Role Stress Scale [31]. This scale consists of five items measuring role ambiguity and eight items that assess role conflict. This instrument uses a seven-point Likert-type scale requiring participants to report on a scale from (1) “totally disagree” to (7) “totally agree”. This scale has shown adequate psychometric properties in previous studies [72]. We used the validated Spanish version [73]. In this study, the Cronbach’s alpha for role ambiguity was 0.79 and for role conflict was 0.88.

#### 2.2.3. Engagement

We used the Utrecht Work Engagement Scale (UWES; [74]). This scale consists of fifteen items grouped into three subscales of work engagement: vigor, dedication, and absorption. This instrument uses a Likert-type scale requiring participants to report on a scale from (0) “never” to (6) “always”. The UWES has shown adequate psychometric properties in previous studies across cultures and occupations [11,75]. We used the Spanish version of the UWES in our study [11]. Given our objectives and hypotheses, in addition to assessing the three engagement dimensions, we created a composite measure of work engagement. Therefore, the scores on the three subscales were converted to z-scores and summed. The Cronbach’s alpha for vigor was 0.84, for dedication was 0.85, and for absorption was 0.82. The Cronbach’s alpha for the three composite work engagement measure was 0.92.

## 3. Results

### 3.1. Descriptive Analyses

The Pearson correlations, means, standard deviations, and reliability of the study variables are shown in Table 1. As can be seen, emotional intelligence was positively and significantly related to vigor, dedication, and absorption and negatively associated with role ambiguity and role conflict. Similarly, role ambiguity/conflict were negatively related to engagement dimensions.

### 3.2. Hierarchical Regression Analysis

We conducted several hierarchical multiple regression analyses in order to examine both the predictive effects of role ambiguity/conflict and EI, together with the potential interactive effect of role ambiguity and role conflict on EI in accounting for variance in work engagement. This method allows the examination of the increment in R^2^ from the addition of another predictor variable into the regression equation. Moreover, this method allows it to be determined whether the added variable explains additional variance not explained by measures previously entered in the model. In all our regression equations, gender, age, grade level taught, and teaching experience were entered in the first step as covariates. In the second step, the overall scores on role ambiguity and role conflict were entered, followed by scores on the WLEIS in the third step. The multiplicative term was entered in the final step of the equation to test the potential moderating between both role ambiguity x EI and role conflict x EI. Therefore, all the continuous predictors were centered in order to reduce the potential problems of multicollinearity [76]. In Table 2, the principal results of these analyses are presented. 

Firstly, for predicting scores on the vigor dimension, a total of 24% of the variance was accounted for (*R*^2^ = 0.236; F (2278) = 9.521; *p* < 0.050). As can be seen in Table 2, after controlling for background variables, role ambiguity and role conflict were found to account for significant variance in predicting vigor (**Δ***R*^2^ = 0.130; *p* < 0.001). In addition, EI was found to explain a significant amount of the variance in vigor (**Δ***R*^2^ = 0.063; *p* < 0.001), even after accounting for the variance attributable to socio-demographic variables and role ambiguity/conflict. Regarding the formulated interactive model, the role ambiguity x EI interaction was found to explain an additional, small but still significant, variance in vigor (**Δ***R*^2^ = 0.019; *p* < 0.050) beyond the variance contributed by the main effects of the teachers’ background characteristics, role ambiguity, role conflict, and EI.

**Figure 1:**
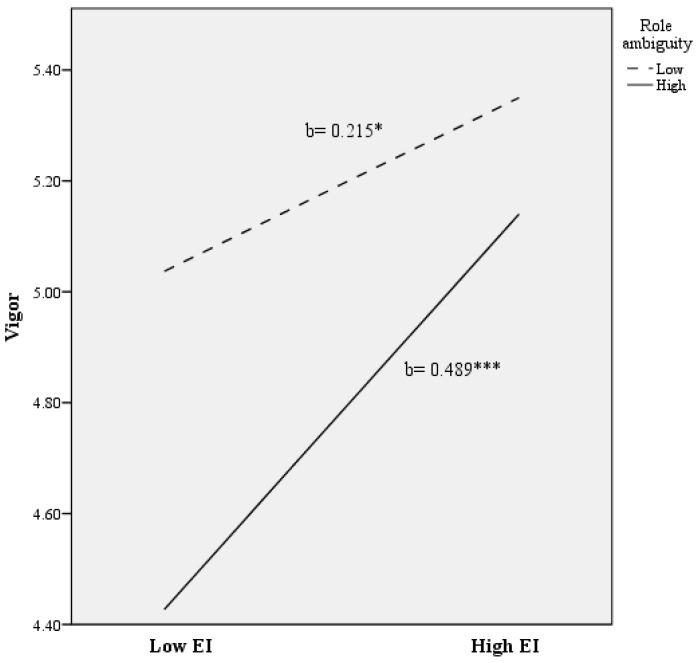
The relationship of emotional intelligence and role ambiguity for predicting vigor scores. Note: EI = Emotional Intelligence. * *p* < 0.050; *** *p* < 0.001.

**Figure 2:**
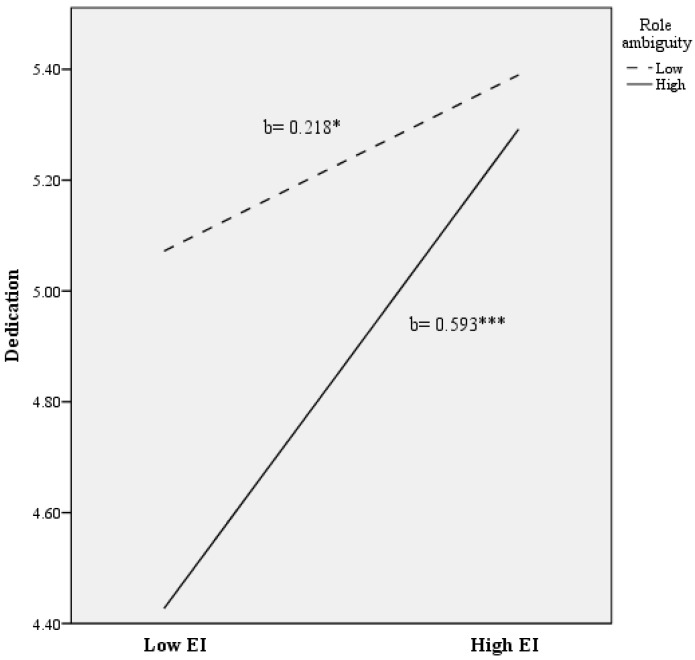
The relationship of Emotional Intelligence and role ambiguity for predicting dedication scores. Note: EI = Emotional Intelligence. * *p* < 0.050; *** *p* < 0.001.

**Figure 3:**
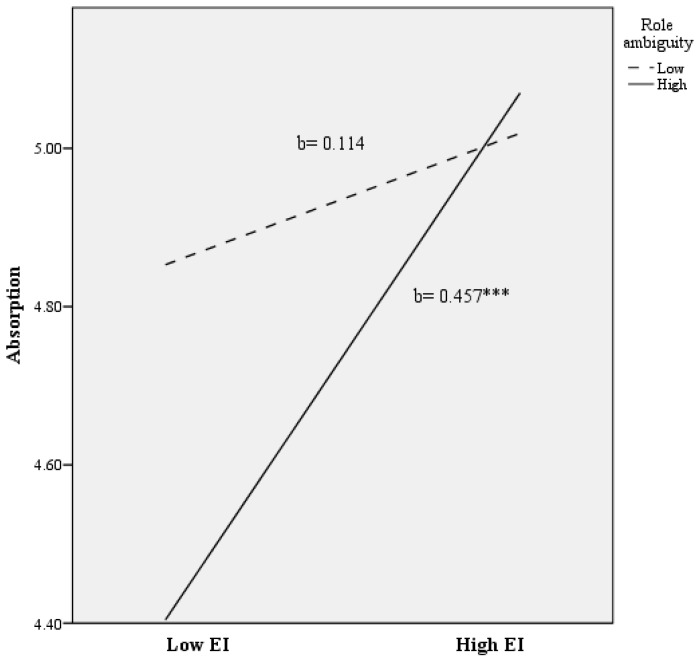
The relationship of emotional intelligence and role ambiguity for predicting absorption scores. Note: EI = Emotional Intelligence. *** *p* < 0.001.

**Figure 4:**
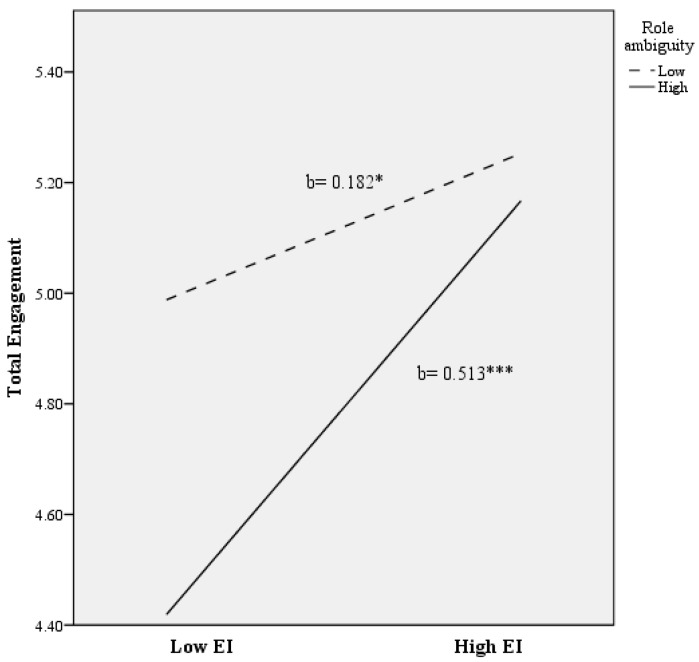
The relationship of emotional intelligence and role ambiguity for predicting total engagement scores. Note: EI = Emotional Intelligence. * *p* < 0.050; *** *p* < 0.001.

To illustrate the role ambiguity x EI interaction for the engagement total score and dimensions, we plotted the regression of UWES on EI at high and low levels of role ambiguity (see Figure 1, Figure 2, Figure 3 and Figure 4). Following the procedures outlined by Aiken and West (1991), we used the simple slope for the regression of the overall UWES scores and subscales on EI by using the high (one standard deviation above the mean) and low (one standard deviation below the mean) values for role ambiguity [76]. As is shown in Figure 1, there was a significant positive association between EI and vigor at lower levels of role ambiguity ((b = 0.215), t (278) = 2.337; *p* < 0.050). Nevertheless, a more intense association between EI and vigor was found at higher levels of role ambiguity ((b = 0.489), t (278) = 5.177; *p* < 0.001). 

Secondly, for predicting scores on dedication, a total of 24% of the variance was accounted for (*R*^2^ = 0.239; F (2278) = 9.711; *p* < 0.010). As Table 2 shows, after controlling for the teachers’ background characteristics, role ambiguity and role conflict were found to account for significant variance in predicting dedication (**Δ***R*^2^ = 0.104; *p* < 0.001). In addition, EI was found to explain significant additional variance in dedication (**Δ***R*^2^ = 0.077; *p* < 0.001), even after controlling for the variance attributable to socio-demographic variables and role ambiguity/conflict. Regarding the formulated interactive model, it is noteworthy that the role ambiguity x EI interaction was found to explain additional variance in dedication (**Δ***R*^2^ = 0.026; *p* < 0.010) beyond the main effect of the covariates, role ambiguity, role conflict, and EI. As Figure 2 shows, there was a significant positive association between EI and dedication at lower levels of role ambiguity ((b = 0.218), t (278) = 2.225; *p* < 0.050). Nevertheless, a more intense association between EI and dedication was found at higher levels of role ambiguity ((b = 0.593), t (278) = 5.886; *p* < 0.001).

Thirdly, a total of 14% of the variance in absorption scores was predicted (*R*^2^ = 0.143; F (2278) = 5.163; *p* < 0.050). As is shown in Table 2, after controlling for age, gender, grade level taught, and teaching experience, role ambiguity and role conflict were not found to account for significant variance in predicting absorption. Nonetheless, EI was found to account for a significant additional percentage of the variance in absorption subscale (**Δ***R*^2^ = 0.032; *p* < 0.010), after accounting for the variance attributable to covariates and role ambiguity/conflict. Finally, the EI x role ambiguity interaction was found to explain an additional variance in absorption (**Δ***R*^2^ = 0.019; *p* < 0.050) beyond the variance contributed by the main effects of the background characteristics, role ambiguity, role conflict, and EI. As is shown in Figure 3, the association between EI and absorption at lower levels of role ambiguity was non-significant ((b = 0.114), t (278) = 1.052). On the other hand, we found that EI and absorption were significantly and positively related at higher levels of role ambiguity ((b = 0.457), t (278) = 4.091, *p* < 0.001).

Finally, for predicting scores on overall engagement, a total of 25% of the variance was accounted for (*R*^2^ = 0.245; F (2278) = 10.03; *p* < 0.010). As Table 2 shows, after controlling for teachers’ background characteristics, role ambiguity and role conflict were found to account for significant variance in predicting work engagement (**Δ***R*^2^ = 0.110; *p* < 0.001). In addition, EI was found to explain significant additional variance in engagement (**Δ***R*^2^ = 0.070; *p* < 0.001), even after controlling for the variance attributable to socio-demographic variables and role ambiguity/conflict. Moreover, it is noteworthy that the role ambiguity x EI interaction was found to explain additional variance in overall engagement (**Δ***R*^2^ = 0.026; *p* < 0.010) beyond the main effect of the covariates, role ambiguity, role conflict, and EI. As Figure 4 shows, there was a significant positive association between EI and overall engagement at lower levels of role ambiguity ((b = 0.182), t (278) = 2.120; *p* < 0.050). Nevertheless, a more intense association between EI and engagement was found at higher levels of role ambiguity ((b = 0.513), t (278) = 5.799; *p* < 0.001).

## 4. Discussion

This study expands on previous research in the literature on EI and engagement [54,58,67] by examining the unique and interactive effects of EI and role stress in accounting for engagement in a sample of teachers. Firstly, our findings showed that teachers with higher scores in EI reported less stress arising from ambiguous information within teaching contexts, as well as less stress arising from contradictory information at work. Consistent with previous studies [40,69], these results highlight the key role of EI in reducing occupational stress among teachers [59,68,77]. As in prior research, we found significant positive associations between EI and engagement dimensions [58,66,67]. Specifically, we found that EI positively accounted for engagement scores, even after controlling for background characteristics such as teachers’ age, gender, teaching experience, and grade level taught. Lastly, in accordance with previous findings, role ambiguity and conflict were found to be significantly and negatively linked to work engagement [16,39,40]. Our results particularly showed that role ambiguity negatively accounted for a statistically significant portion of the variance in the overall engagement, vigor, and dedication scores, whereas role conflict did not account for work engagement.

### 4.1. Interactive Contribution of Work Environment Characteristics and EI

With respect to the interactive contribution of role ambiguity/conflict and EI for predicting teachers’ engagement, our results showed that the interaction between role ambiguity and EI significantly augmented the prediction of vigor, dedication, and absorption scores beyond the main effects of these constructs. We found a similar pattern with respect to overall engagement. More interestingly, although role ambiguity did not show major effects on the absorption levels, when examined along with EI, the resultant interactive effect showed significant augmentation in accounting for absorption scores. This differential pattern appears to be in line with previous studies, suggesting that vigor and dedication constitute the core of engagement [3,9]. Given that role stress has shown differential patterns influencing teacher engagement through time [40], these findings might have implications for further studies aiming to examine the concurrent influence of organizational factors and personal resources such as EI.

When analysing the interaction effects of role ambiguity and EI in predicting teacher engagement, the results showed that the magnitude of the association between EI and the engagement dimensions was significantly greater in the presence of high role ambiguity than low role ambiguity scores. In this line, previous studies have shown that job resources become more salient in a context of resource loss [78,79]. For instance, it has been found that the influence of job resources on engagement is particularly high when teachers are confronted with higher levels of pupil misconduct [78]. Similarly, Seers, McGee, Serey, and Graen (1983) found that social support was inconsequential for predicting job satisfaction when the employees did not experience role conflict, whereas those who suffered from higher stress caused by role conflict used social support in order to achieve more positive work attitudes [80].

Regarding the most recent review of the JD-R model [13,42], prior research has shown that the influence of personal resources on work engagement can be particularly significant in the presence of high demands [20,43]. A longitudinal study with newly qualified teachers has provided evidence on the salience of personal resources in relation to teacher engagement when classroom disturbances were higher [20]. The interaction effect referred to as the “boosting effect” of job demands on the associations between both job and personal resources and engagement has been increasingly reported [20,43,81]. Similarly, applying the situation-specific model described by Côté (2014), the magnitude of associations between EI and teacher engagement would vary depending on organizational factors, that is, levels of role ambiguity [46]. One plausible explanation of these and previous findings has been proposed in terms of the Conservation of Resources Theory [82]; it has been argued that a context with high demands can lead to the particular salience of resources, which therefore strongly influence engagement [78,79]. Consistent with this reasoning, teachers appear to be more likely to need to use their affective processing resources when dealing with stress related to ambiguous duties, tasks, and responsibilities [20,43].

### 4.2. Limitations 

When interpreting the contributions of the current study, possible limitations should be considered. First, the cross-sectional design used in our work limits the determination of any causal relationship between variables. In this sense, elucidating how job demands and EI influence engagement through time is a promising approach, and, hence, prospective analyses are required to test the relations between these variables [20,40]. Moreover, such relations should be tested at the within-person level using experience-sampling designs [18]. Second, the inclusion of performance-based EI tests is crucial in order to examine the relationship between EI and engagement [44]. Third, although our study showed interaction effects between role ambiguity and EI in predicting work engagement, role conflict did not interact with EI in accounting for engagement. True interaction effects are typically difficult to detect in non-experimental designs because of limited statistical power [83], and, hence, relatively large samples are required for the effects to be significant. Although the current sample size provided adequate statistical power to detect interaction effects, future studies with higher samples are advised to examine this issue in depth [84,85]. Likewise, these findings need to be replicated with larger and more heterogeneous teacher populations in order to increase the generalizability our results [77]. Additional research should also examine the differential role of job hindrances and job challenges when examining their potential boosting effects on the resources-work outcomes associations [43]. Finally, it should be noted that, compared to the main effects, the percentage of explained variance attributable to the role ambiguity x EI interaction in predicting the overall engagement and dimensions was very modest (from 0.02 to 0.03). Nonetheless, researchers have argued that even a 1% contribution of the total variance should be considered particularly noticeable as the efficiency of the estimation of these interactions is primarily low [84].

### 4.3. Implications for Further Research

Despite the limitations we noted above, there are important contributions in this study. Prior research has shown the independent associations of EI and role stress on engagement [16,39,40]. However, in the case of teacher engagement, this is the first study of which we are aware to empirically test the joint contribution of EI and role ambiguity in explaining this work outcome. Additionally, our findings are valuable in designing more integrative models, considering the impact of job demands on the associations between personal resources such as EI, engagement, and commitment to teaching [28,36,86]. Likewise, personality traits should be examined in future research due to their effects on occupational well-being [87] and work attitudes [35,88]. Finally, because reciprocal associations between personal resources and work engagement have been reported [20,89], this line of enquiry merits increased attention. 

The practical implications of our results are particularly relevant in relation to the design of training courses for teaching professionals. For instance, practitioners might monitor teachers with low EI levels, when they are in situations with high role stress, as these professionals are at especially high risk of experiencing low levels of energy and dedication and to be less immersed in their teaching work. In addition, using personnel selection methods based on EI and designing a supportive workplace environment to ensure a good balance between individual teachers’ resources and organisational characteristics would help to increase their work engagement. Our findings suggest possible avenues for improving future intervention programs in order to promote occupational well-being among teachers. While organization-focused interventions are important in achieving occupational well-being in educational settings [4,6], the current study shows preliminary evidence for the importance of paying more attention to the interactive effects of contextual and personal factors. In this sense, a recent meta-analytic review on the effectiveness of EI training has suggested identifying specific situational factors as a promising approach [90]. Relatedly, a meta-analytic review on work engagement interventions supports the notion that EI training in educational settings aiming to increase teachers’ resources is required [67,91]. Finally, the consideration of work-related stressors appears to be key in designing EI training directed to the enhancement of occupational well-being. This could, in turn, have effects on educators’ work attitudes, aiming to alleviate the crisis of teacher attrition [40,92]. 

## 5. Conclusions

In summary, it appears critical to examine the impact of job demands in explaining teachers’ levels of engagement beyond the influence of personal resources such as EI. Future studies directed toward the influence of organizational factors on the association between emotional abilities and engagement represent a promising approach to further understanding how the positive aspects of work attitudes among teachers can be promoted.

## Figures and Tables

**Table 1:** The means, standard deviations, reliabilities, and correlations between study variables.

Variables	1	2	3	4	5	6	7
1. Emotional Intelligence	-						
2. Role Ambiguity	−0.405 **	-					
3. Role Conflict	−0.187 **	0.205 **	-				
4. Vigor	0.390 **	−0.365 **	−0.183 **	-			
5. Dedication	0.397 **	−0.336 **	−0.117 *	0.750 **	-		
6. Absorption	0.279 **	−0.217 **	−0.137 *	0.610 **	0.677 **	-	
7. Total Engagement	0.401 **	−0.345 **	−0.164 **	0.887 **	0.913 **	0.860 **	-
Mean	5.39	2.49	3.46	4.95	4.99	4.79	4.91
SD	0.73	0.94	1.44	0.88	0.94	0.98	0.83
Cronbach’s α	0.91	0.80	0.87	0.84	0.85	0.82	0.92

*N* = 288. * *p* < 0.050, ** *p* < 0.010.

**Table 2:** Hierarchical regression analysis showing the amount of variance in engagement accounted for by role stressors and emotional intelligence (EI) interactions.

Predictors	Vigor				Dedication				Absorption				Total Engagement			
	*R*^2^	*F*	*β*	Δ*R*^2^	*R*^2^	*F*	*β*	Δ*R^2^*	*R*^2^	*F*	*β*	Δ*R*^2^	*R*^2^	*F*	*β*	Δ*R*^2^
**Step 1**	0.023	1.673		0.023	0.032	2.368		0.032	0.05	3.724		0.05 **	0.039	2.844		0.039 *
Age			0.06				0.009				−0.139				−0.026	
Gender			0.053				0.068				0.049				0.064	
Grade level taught			−0.03				−0.042				−0.095				−0.063	
Teaching experience			−0.044				−0.091				0.154				0.007	
**Step 2**	0.153	8.462		0.13 ***	0.136	7.401		0.104 ***	0.092	4.723		0.042 **	0.148	8.155		0.11 ***
Role ambiguity			−0.227 ***				−0.20 ***				−0.088				−0.194 ***	
Role conflict			−0.095				−0.03				−0.079				−0.076	
**Step 3**	0.216	11.036		0.063 ***	0.214	10.859		0.077 ***	0.124	5.649		0.032 **	0.219	11.23		0.07 ***
EI			0.282 ***				0.311 ***				0.202 ***				0.299 ***	
**Step 4**	0.236	9.521		0.019 *	0.239	9.711		0.026 **	0.143	5.163		0.019 *	0.245	10.03		0.026 **
Role ambiguity x EI			0.116 *				0.142 **				0.143 *				0.151 **	
Role conflict x EI			0.062				0.057				−0.013				0.039	

Note: EI = Emotional Intelligence. The beta reported in the table is the standardized regression coefficient for the final equation. * *p* < 0.050, ** *p* < 0.010, *** *p* < 0.001.
